# Local patient-centered headache advocacy with regional and global impact: lessons from the Japanese Patient Advocacy Coalition (JPAC)

**DOI:** 10.1186/s10194-026-02302-5

**Published:** 2026-02-23

**Authors:** Daisuke Danno, Eriko Yamanaka, Kaori Tabata, Shoji Kikui, Min Kyung Chu, Olivia Begasse de Dhaem, Koichi Hirata, Fumihiko Sakai, Takao Takeshima

**Affiliations:** 1 Headache Center, Department of Neurology, Tominaga Hospital, Osaka, Japan; 2 Japanese Patient Advocacy Coalition (JPAC), Tokyo, Japan; 3 Department of Neurology, Severance Hospital, Yonsei University College of Medicine, Seoul, Korea; 4 University of Connecticut, Farmington, CT USA; 5 Global Patient Advocacy Coalition for Headache (GPACH), Brussels, Belgium; 6 Department of Neurology, Dokkyo Medical University, Mibu, Japan; 7 Saitama International Headache Center, Saitama, Japan

**Keywords:** Migraine, Patient advocacy, Patient awareness, Patient education, Health professional education, Stigma, Japan, Asia-Pacific collaboration, GPACH, IGAP

## Abstract

Migraine is a leading cause of disability worldwide, particularly among working-age individuals. In Japan, its prevalence is 8.4%; however, migraine remains underdiagnosed and undertreated. Only 39.7% of patients with migraine seek care for it and 9.2% receive preventive care. Migraine stigma is ubiquitous and concealment has become a trait of the disease, highlighting the urgent need for public awareness and patient advocacy. The Japanese Patient Advocacy Coalition (JPAC) was established in 2017 following the inaugural Global Patient Advocacy Summit in Vancouver, which aimed to place patients at the center of advocacy and address their needs worldwide. With the support of the Japanese Headache Society (JHS) and in alignment with the Global Patient Advocacy Coalition (GPAC, now GPACH), JPAC promotes awareness, reduces stigma, and amplifies the voices of patients in Japan. Since its inception, the JPAC has led various initiatives nationwide to raise awareness, provide education, promote advocacy, and break down stigma. To raise awareness and increase understanding, education initiatives have targeted the public, patients, nurses, healthcare professionals, hospital staff, occupational physicians, and workplaces. JPAC has also led to successful international collaboration. Expanding JPAC initiatives to schools, workplaces, and policy settings will be essential for reducing stigma and improving the lives of individuals living with headache disorders. JPAC’s collaborative, patient-centered efforts, anchored by individuals living with headache disorders and supported by the JHS, have significantly advanced awareness and advocacy in Japan. This manuscript reviews eight years of JPAC’s local initiatives and the lessons learned, aiming to serve as a blueprint, source of inspiration, and support for headache advocacy efforts regionally and globally.

## Introduction

More than 3 billion people worldwide have a neurological disorder, which represents approximately 43% of the world’s population and is associated with high levels of disability. Migraine is the leading cause of disability-adjusted life years (DALYs) among individuals of 5–19 years of age and the second leading cause among those of 20–59 years of age. Migraine imposes a substantial burden on patients’ lives and accounts for more than 43 million years lived with disability [1]. The prevalence of migraine in Japan was estimated at 8.4% based on the first nationwide survey, reported in 1997. Headache frequency is associated with disability, similar to that observed in other regions of the world. The proportion of Japanese patients with migraine with moderate-to-severe headache-related disability (Migraine Disability Assessment total score [MIDAS] ≥ 11), based on a cross-sectional analysis of real-world clinical data collected in Japan in 2014, was 25.5% for those with 4–7 monthly headache days (MHDs), 41.1% for those with 8–14 MHDs, and 60.0% for those with at least 15 MHDs [2, 3]. Furthermore, a workplace survey conducted in Japan in 2018 reported that migraine significantly reduced employee productivity and quality of life, with per-person economic losses estimated at $238.3 per year due to absenteeism and $2,217 per year due to presenteeism. In Japan, the nationwide productivity loss related to migraine-associated presenteeism was estimated at $21.3 billion annually [4]. Despite being a highly disabling disease, migraine has remained underdiagnosed and undertreated in Japan. Japan has a population of over 120 million and a universal health insurance system under which most medications, including Calcitonin Gene-Related Peptide monoclonal antibodies (CGRP-mAbs), are partially reimbursed. The Japanese Headache Society (JHS) certifies headache specialists, with 978 physicians currently accredited; however, a population-based web survey conducted in Japan (the OVERCOME Japan study, published in 2021) found that only 39.7% of people with migraine sought medical care in the past year, and only 9.2% received preventive medication [5]. Reasons reported not seeking medical care for headaches included being able to endure symptoms without a doctor’s consultation or medication, effective treatment of headaches with over-the-counter (OTC) drugs, and inability to take time off work for treatment [6], suggesting limited awareness of the potential risk of headache chronification and highlighting the need for workplace interventions.

In terms of migraine-related stigma (MiRS), a population-based web survey conducted in Japan between July and September 2020 found that 47.2% of employed individuals with migraine felt that their employers were not highly understanding of their condition, 16.5% frequently experienced stigma, and approximately 30–40% reported hiding their migraine from others. Migraine-related stigma has been shown to be common and strongly associated with greater disability, interictal burden, and reduced quality of life in large population-based studies, underscoring the global need for patient advocacy [7]. From these findings, it was speculated that while stigma and social burden exist, they have been underrecognized in Japanese society [8]. This lack of awareness and stigma surrounding migraine highlights the considerable unmet need in the Japanese population, and the need for patient advocacy has been increasing. This review provides a detailed account of the establishment of JPAC in 2017, the subsequent development of its activities, and the lessons learned, including patient-centered initiatives at our headache center. Furthermore, building on JPAC’s role as a model for patient empowerment in headache disorders, we aim to outline the next steps essential for advancing patient-centered advocacy initiatives.

### The Vancouver declaration and the establishment of JPAC

In September 2017, the inaugural Global Patient Advocacy Summit was held in Vancouver, Canada. The summit convened patients, healthcare professionals (HCPs), and other key stakeholders to advance headache care, place patients at the center of advocacy initiatives, and address patients’ needs on a global scale. Participants recognized the importance of addressing diverse patient needs, reducing stigma, ensuring access to competent care, enhancing professional training, establishing global standards, and understanding patient preferences. One of the principal guiding principles was to “think globally and act locally.” Subsequently, a series of statements, including the formation of the International Headache Society Global Patient Advocacy Coalition (IHS-GPAC; currently the Global Patient Advocacy Coalition for Headache [GPACH]), was adopted as the Vancouver Declaration on Global Headache Patient Advocacy 2018 [9].

**Fig. 1:**
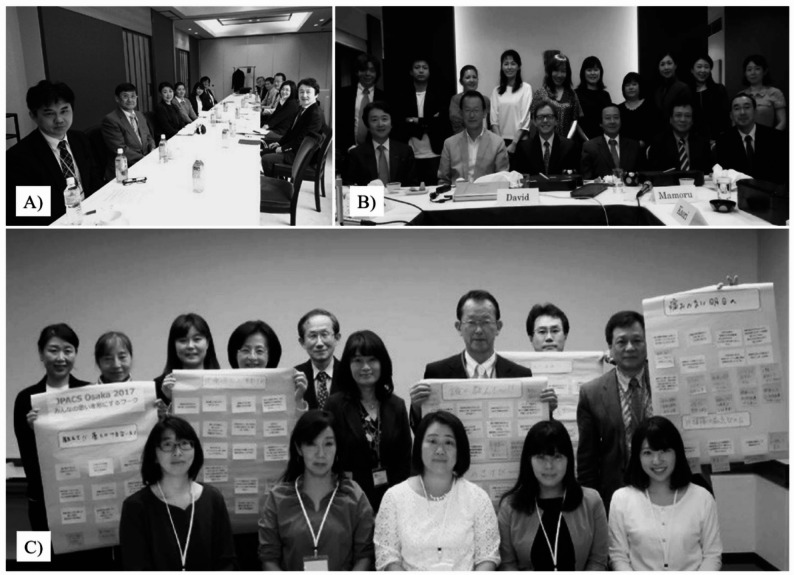
The Foundation of the Japanese Patient Advocacy Coalition (JPAC). An inaugural meeting discussing the quality of life of patients with headache disorders was held in Shizuoka in June 2017 (**A**), followed by a second meeting in Tokyo in September 2017 (**B**). In the third meeting held in Osaka in November 2017, we sought to express our thoughts in words and represent them visually (**C**)

Concurrently in Japan, public awareness campaigns were initiated. The first meeting to discuss the quality of life (QOL) of patients with headache disorders was held in Shizuoka in June 2017, followed by a second meeting in Tokyo in September 2017 (Fig. 1A and B). At the second meeting, Professor Sakai proposed the establishment of the Japanese Patient Advocacy Coalition (JPAC) in alignment with the foundation of the GPAC/GPACH. In response, the Japan Headache Society (JHS) formally launched JPAC activities, co-chaired by Professor Hirata and Nurse Tabata, with the aim of incorporating patient and advocate perspectives regarding stigma, disease awareness, and headache management. JPAC membership includes patients living with headache disorders, advocates, HCPs, and other stakeholders. The Japan Headache Association (JHA), which promotes patient welfare and collaboration through awareness, education, and research, fully supports JPAC activities.

**Fig. 2:**
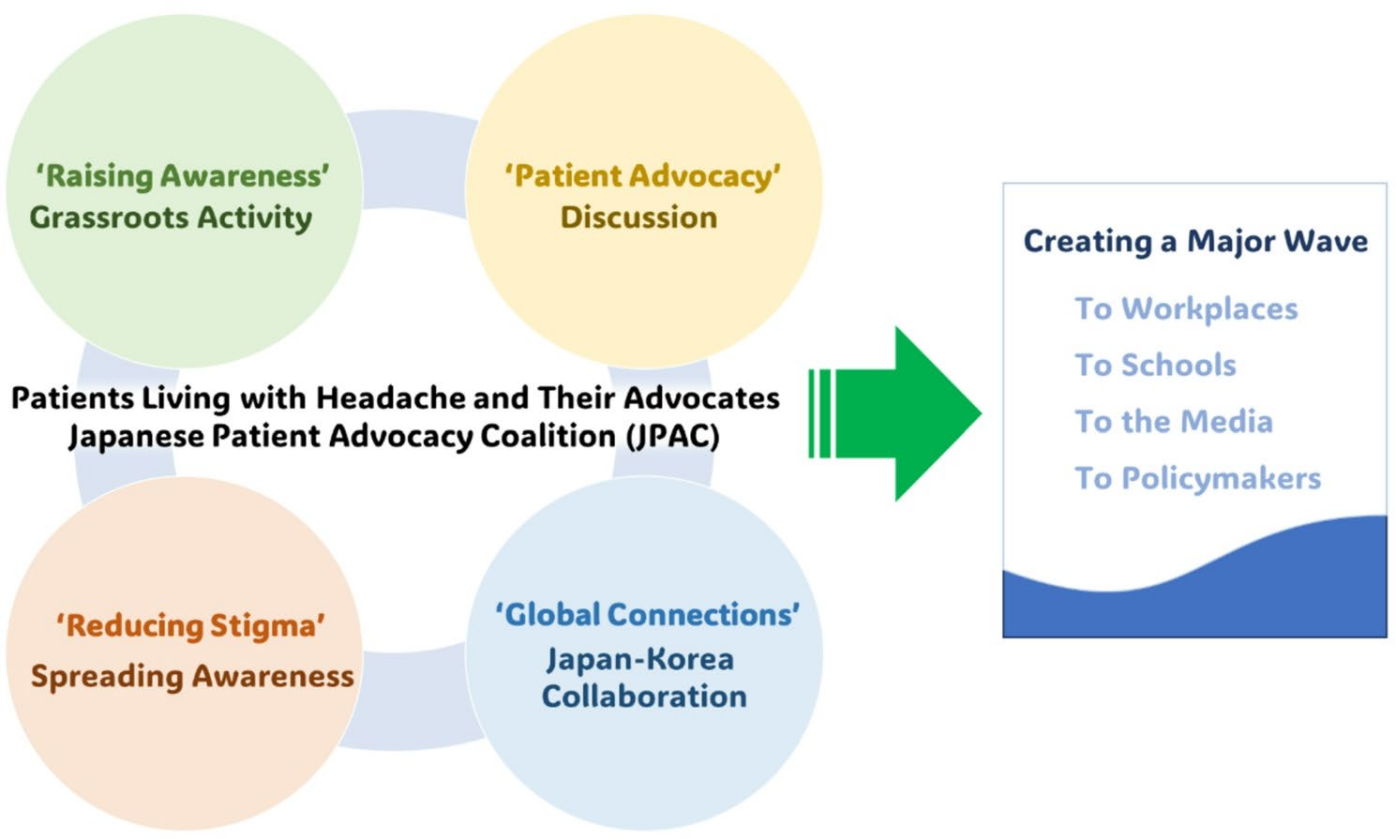
Conceptual Diagram of the Japanese Patient Advocacy Coalition (JPAC). Patient-centered key components—raising awareness, patient advocacy, stigma reduction, and global collaboration on headache disorders—work synergistically to generate a widespread impact across workplaces, schools, the media, and among policymakers

In November 2017 in Osaka, during the 45th Annual Congress of the JHS (President: Professor Takeshima), JPAC held its third meeting and first summit. Patients and HCPs collaborated to organize and categorize their ideas, explore the future direction of advocacy activities, and identify shared goals and priorities. Participants also expressed their perspectives in both written and visual formats to facilitate communication and planning (Fig. 1C). This patient-centered collaborative approach represented a first-of-its-kind initiative in Japan and constituted a critical step in advancing patient advocacy. Since then, JPAC has continued to hold annual summits, promote nationwide grassroots activities, raise awareness, and foster advocacy and stigma reduction through collaborations across Asia. A summary of JPAC’s early activities and a conceptual diagram are presented in Table 1; Fig. 2, respectively.

**Table 1:** Early activities of the Japanese Patient Advocacy Coalition (JPAC)

Date	Event	Location	Notes	Participants
June 18, 2017	1st Meeting: “Discussing QOL of Headache Patients”	Shizuoka	Held during Headache Master School Japan	12 volunteers (patients and healthcare professionals)
September 6, 2017	The Global Patient Advocacy Summit (GPAS)	Vancouver	Core members of the Japanese Headache Society participated	Patients, patient advocates, representatives of patient advocacy organizations, healthcare professionals, pharmaceutical industry representatives, researchers, and regulatory authorities
September 21, 2017	2nd Meeting: “Discussing QOL of Headache Patients”; “Gathering with Prof. Dodick”	Tokyo	Held during the World Congress of Neurology. The name “Japanese Patient Advocacy Coalition (JPAC)” was adopted and activities were officially launched.	15 volunteers (patients and healthcare professionals)
November 11, 2017	3rd Meeting: “Promoting Headache Care: Patients & Doctors”	Osaka	Held during the 45th Annual Meeting of the Japanese Headache Society. JPAC convened its third meeting in this series in Osaka, marking the first summit format. Group work titled “Turning Everyone’s Thoughts into Action.”	17 volunteers (patients and healthcare professionals)
November 16, 2018	1st JPAC Public Lecture for Citizens	Kobe	Held during the 46th Annual Meeting of the Japanese Headache Society	The general public

#### Synergy in collaboration: JPAC, JHS, patients, and HCPs

The JHS is a professional organization primarily composed of HCPs and researchers, supporting high-quality clinical practice through the dissemination of current guidelines and research evidence. JPAC aggregates perspectives from patients and advocates, provides patient-centered support, and engages in awareness-raising activities. Collaboration between these entities integrates evidence-based knowledge with JPAC’s access to patient communities and real-world insights, thereby enhancing public understanding and promoting accurate dissemination of information.

In the development of the Japanese Clinical Practice Guideline for Headache Disorders, patient representatives participate alongside HCPs, ensuring that patient perspectives inform recommendations [10]. In addition, JPAC conducts surveys following its various initiatives to reflect patient feedback in subsequent events. Integrating patient voices and experiences into clinical guidelines and these initiatives is expected to improve the quality of patient-centered care. This collaborative framework also has the potential to strengthen legislative advocacy and optimize healthcare resource allocation, thus forming a vital foundation for the advancement of headache medicine.

### JPAC’s model of patient-centered advocacy

Similar to JPAC, there are other patient-centered advocacy organizations that collaborate with professional organizations around the world.

In the United States of America, the American Migraine Foundation (AMF) has developed a large national patient community through digital initiatives, including the “Move Against Migraine” Facebook support group and the online “Migraine Advocacy Hub.” AMF provides education, support and opportunities for advocacy for people living with migraine in close collaboration with the American Headache Society, the professional headache organization [11]. In the United Kingdom, The Migraine Trust (MT) is a national charity that aims to empower, inform and support people affected by migraine, while educating health professionals, funding migraine research and engaging in policy initiatives to ensure that migraine is appropriately recognized on the health agenda [12].

In this context, JPAC can likewise be positioned as a hybrid collaborative model that maintains organizational independence while working in close partnership with JHS and receiving structural and sustained support. JPAC’s activities are jointly carried out by a diverse group of stakeholders, including individuals living with headache disorders, and the themes and priorities of advocacy are defined based on patients’ needs and perspectives. These activities are consistently implemented through a bottom-up, grassroots approach grounded in engagement with local communities. Through this organizational structure, JPAC maintains patient-centeredness while simultaneously accumulating professional expertise and bridging it to social and institutional contexts. One important feature of JPAC is the active involvement of front-line HCPs engaged in everyday clinical practice, which facilitates direct linkage between patients’ experiences and the realities of clinical care. This feature enables advocacy initiatives to extend beyond conceptual recommendations and develop into practical, clinically relevant, and effective educational and awareness-raising activities. A particularly important feature of JPAC is its close collaboration with GPACH and with HCPs and patients across the Asian region, in line with the principle of “Think globally, act locally.” Through this framework, JPAC conducts advocacy activities rooted in local contexts while aligning them with international knowledge and global networks. This approach enables bidirectional sharing and circulation of experiences, outcomes, and challenges between local practice and global initiatives.

### Raising awareness and providing education at all levels in all settings

To enhance awareness of headache disorders, JPAC has identified the dissemination of accurate evidence-based information as a fundamental objective. The target population extends beyond patients and advocates to include HCPs engaged in direct patient care such as nurses, rehabilitation staff, and allied health workers. Occupational health professionals responsible for managing employee well-being in workplace settings are also regarded as essential stakeholders. Of particular note is a headache project targeting employers of an information technology company. Moreover, to facilitate grassroots activities, JPAC has prioritized patient-centered discussion sessions as a key strategy. The following sections detail the specific strategic measures undertaken by JPAC to further raise awareness and provide education at all levels in all settings.

#### Patient-centered headache class initiatives

**Fig. 3:**
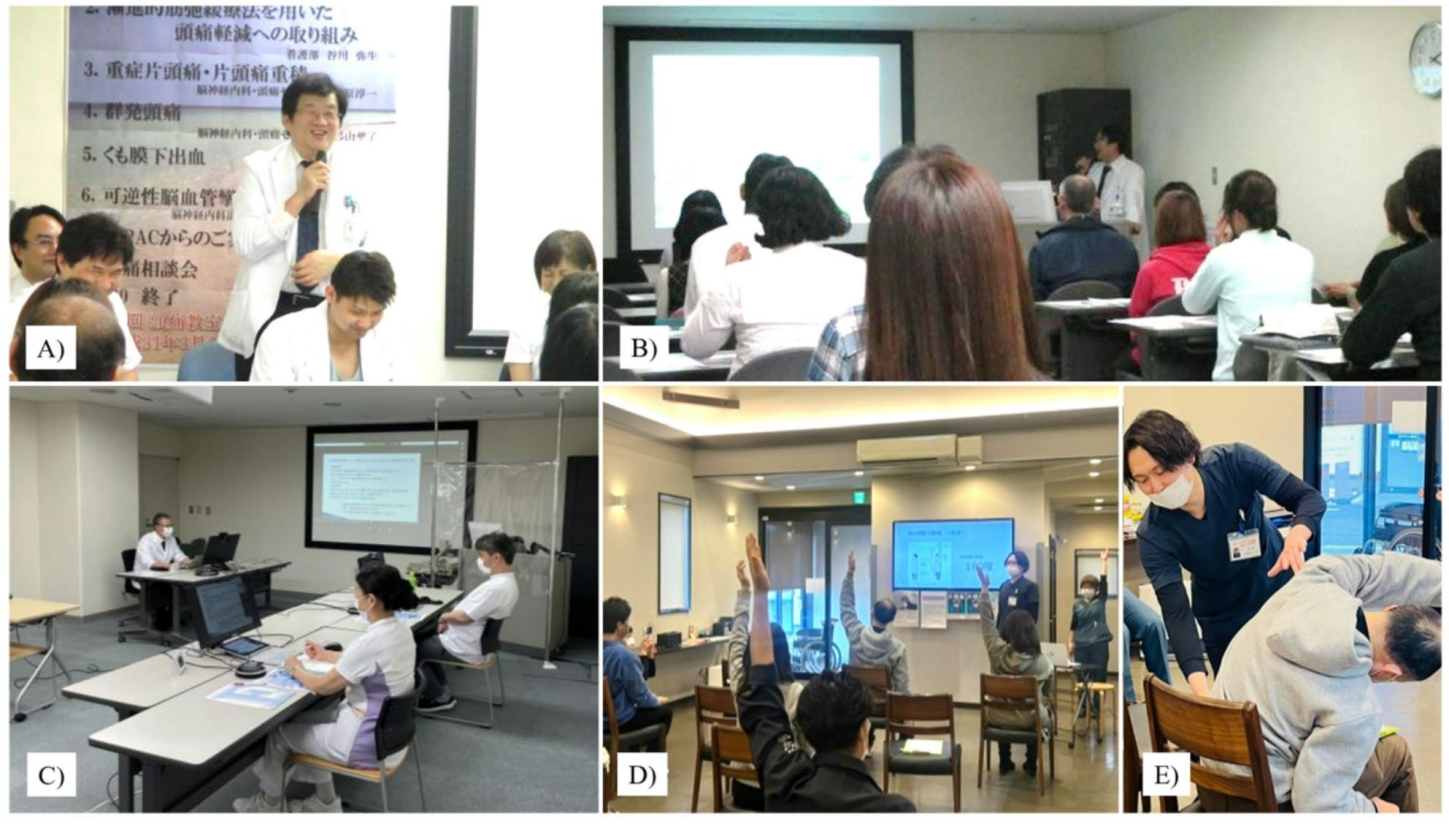
Educational Headache Classes. Headache classes for People Living with Headaches at the Tominaga Hospital Headache Center in 2018 (**A**, **B**). During the COVID-19 pandemic, these classes were held online (**C**). Another program, called “Headache Lab,” was conducted at the Sendai Headache and Neurology Clinic (**D**, **E**)

In response to patients’ demands for more comprehensive information and understanding of their headache disorders, headache classes have been conducted two to three times annually since July 2014 at a tertiary headache center, the Tominaga Hospital Headache Center, with a total of 28 sessions held to date, with approximately 1,400 patients and their caregivers in attendance. The primary objective of these classes is to provide patients with thorough education on headache disorders [13]. They were facilitated by a multidisciplinary team composed of headache specialists, specialized nurses, psychologists, pharmacists, physiotherapists, and nutritionists. These sessions offer patients and advocate opportunities to obtain up-to-date information and enhance their understanding of headache disorders. These sessions are also characterized by active engagement through question-and-answer discussions, which have been reported to significantly enhance patient satisfaction. (Figure 3A and B). Following the COVID-19 pandemic, an online headache education class was initiated and subsequently developed into a hybrid format (Fig. 3C). This hybrid approach has facilitated increased patient participation and enabled the implementation of multicenter joint headache classes, including international collaboration. JPAC provides a range of grassroots activities across Japan aimed at educating and supporting patients. In addition to conventional headache classes at the Tominaga Hospital tertiary headache center, several other centers or clinics organize patient-centered events in the form of roundtable discussions under unique names, including “Headache Peer,” “Headache Lab,” and “Headache Café” (Fig. 3D and E). These events are regularly conducted at leading headache centers, including the Saitama International Headache Center, Sendai Headache and Neurology Clinic, and Japanese Red Cross Society Shizuoka Hospital. In each event, patients and advocates have the opportunity to deepen their understanding of headache disorders while sharing common challenges with fellow attendees, who face similar physical and emotional suffering. These programs were disseminated through multiple channels, including the distribution of flyers at the outpatient clinic, postings within the hospital, announcements on the hospital’s official website, and email notifications sent to previous participants.

#### Training program for headache nurses to further refine advocacy

**Fig. 4:**
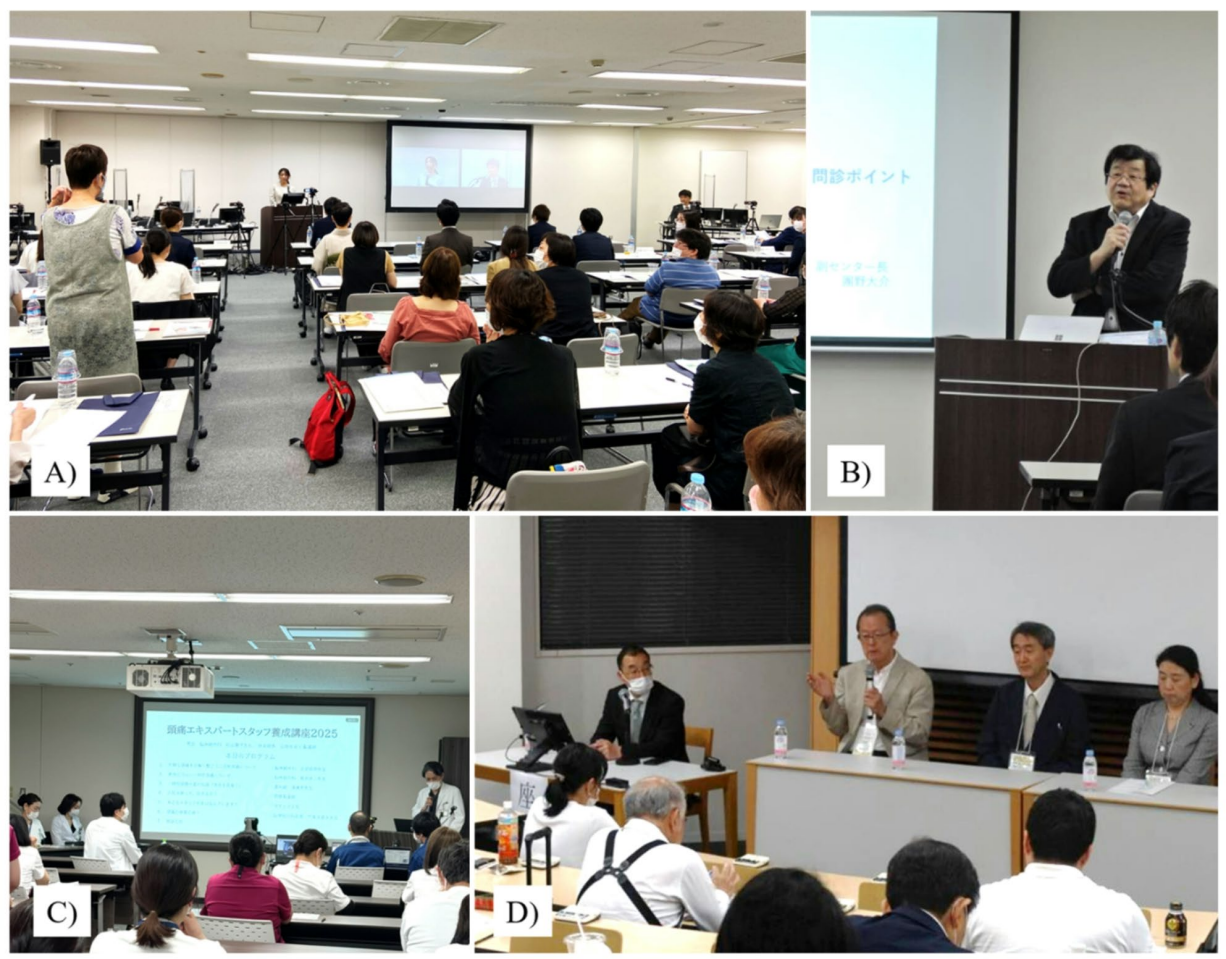
Training Program for Headache Nurses and Other Grassroots Activities. Hybrid training program for headache nurses held in Osaka (**A**, **B**). Training program for staff at the Tominaga Hospital Headache Center (**C**). Educational lectures on headache disorders for occupational physicians, endorsed by the JPAC (**D**)

The roles of headache nurses center around two main themes: “their roles and tasks in the clinical setting” and “their roles and tasks in educating patients and colleagues” [14]. With increasing attention to headache disorders in Japan, the importance of headache nurses has been rapidly and widely recognized. This recognition extends beyond their involvement in supporting the administration of CGRP-mAbs to encompass their vital contribution within patient advocacy and multidisciplinary treatment teams. In this context, the Tominaga Hospital Headache Center launched its inaugural Training Program for Headache Nurses in August 2019. Since then, four sessions have been held (Fig. 4A and B). Physicians and HCPs affiliated with JPAC served as faculty members for the 2024 Headache Nurse Training Course held in Osaka, which was attended by over 500 nurses including online participants. This program provided a valuable platform for attendees to exchange experiences and perspectives, inspiring them to strive toward ideal headache care.

The wide participation—from nurses in small clinics to those in large university hospitals—reflects not only the strong interest in headache care but also the limited opportunities currently available for education on headache disorders. The rapid recognition of the importance of headache nurses in Japan followed the launch of CGRP-mAbs and was fostered through various congresses, seminars, and lectures. Building on this momentum, from the second session onward, a pharmaceutical company that shared JPAC’s vision supported the promotion and dissemination of the sessions.

While healthcare systems and clinical settings vary across countries and even individual institutions [14], this initiative serves as a model for advancing advocacy in headache care.

#### Training program for hospital staff

JPAC offers a training program for Tominaga Hospital staff to raise awareness and increase the understanding of headache disorders in the workplace (Fig. 4C). This program was designed for all hospital staff, including reception clerks. It includes lectures on headache disorders and opportunities for staff to reflect on and share their own experiences with headaches from the patient’s perspective. Through this program, participants gained insight into the burden of headache disorders and learned effective ways to communicate with headache patients. To date, three sessions have been held, with a total of approximately 120 staff members in attendance.

Staff feedback collected after each session highlighted the common tendency, even among nurses, to underestimate headache disorders, reaffirming the importance of creating an environment in which patients can easily communicate their headache disorders and receive support from those around them.

#### Workplace education and management programs

Migraine peaks during people’s working years and is associated with occupational burnout, highlighting the need for workplace supports and adjustments [15]. In a study conducted among a socially active population working in the Tokyo metropolitan area, 22.4% of individuals with migraine reported being obliged to miss work because of headaches several times a year [16]. It has also been reported that economic losses due to presenteeism among employees with migraine exceed those related to absenteeism. These findings highlight the critical need to develop and implement workplace programs for better migraine management in occupational settings [4]. From this perspective, JPAC endorsed an educational lecture on headache disorders for occupational physicians. JPAC members provided a training program accredited by the Japan Medical Association (Fig. 4D). This program facilitated an understanding of the importance of workplace adjustments for sustained performance and was enriched by active discussions, making it a productive session overall. Furthermore, the first organization-wide, large-scale migraine education and management program in the workplace in Japan was the Fujitsu Headache Project—developed in collaboration with JPAC, GPAC (now known as GPACH), and Fujitsu, a leading Japanese company specializing in information and communications technology—which served as a pioneering initiative. The program was offered to all Fujitsu employees based in Japan and achieved exceptionally high participation, with 73,432 employees (90.5%) completing it. Despite the majority of employees being male, the prevalence of migraine was high (16.7%). It followed prior disease-specific health promotion programs in the workplace and was advertised throughout the organization with support from the leadership. In addition to being well-attended, the education program was impactful: 91% of attendees found it useful or very useful, and 73% of attendees increased their understanding of headache disorders (the most common reason for lack of increased understanding was already having had a good understanding). In addition, as a result of participation in the education program, 83% of attendees without headaches said they would change their attitude towards colleagues with headache disorders [17]. The Fujitsu Headache Project is a rare large-scale headache advocacy effort with clearly measured outcomes, and the quantification of awareness improvement achieved through the project is particularly noteworthy, establishing it as a potential model for future awareness-raising initiatives. The results led to the recommendation to create workplaces supportive of workers with headache disorders in the Intersectoral Global Action Plan on Epilepsy and other Neurological Disorders (IGAP) toolkit [18].

### Promoting patient advocacy in underserved areas

As of 2023, there are 978 headache specialists certified by the JHS in Japan. However, the number of specialists per prefecture varies significantly, ranging from 2 in Akita Prefecture to 163 in Tokyo Metropolis, resulting in a problematic uneven distribution [19]. These regional disparities in the distribution of headache specialists pose significant challenges not only to the equitable provision of patient care but also to regional development and the sustainability of patient advocacy efforts. In particular, limited access to specialists in underserved areas can diminish advocacy momentum; therefore, efforts to raise awareness and promote advocacy in such areas are warranted. Addressing this issue is critical for promoting equity in both healthcare delivery and patient representation across Japan.

**Fig. 5:**
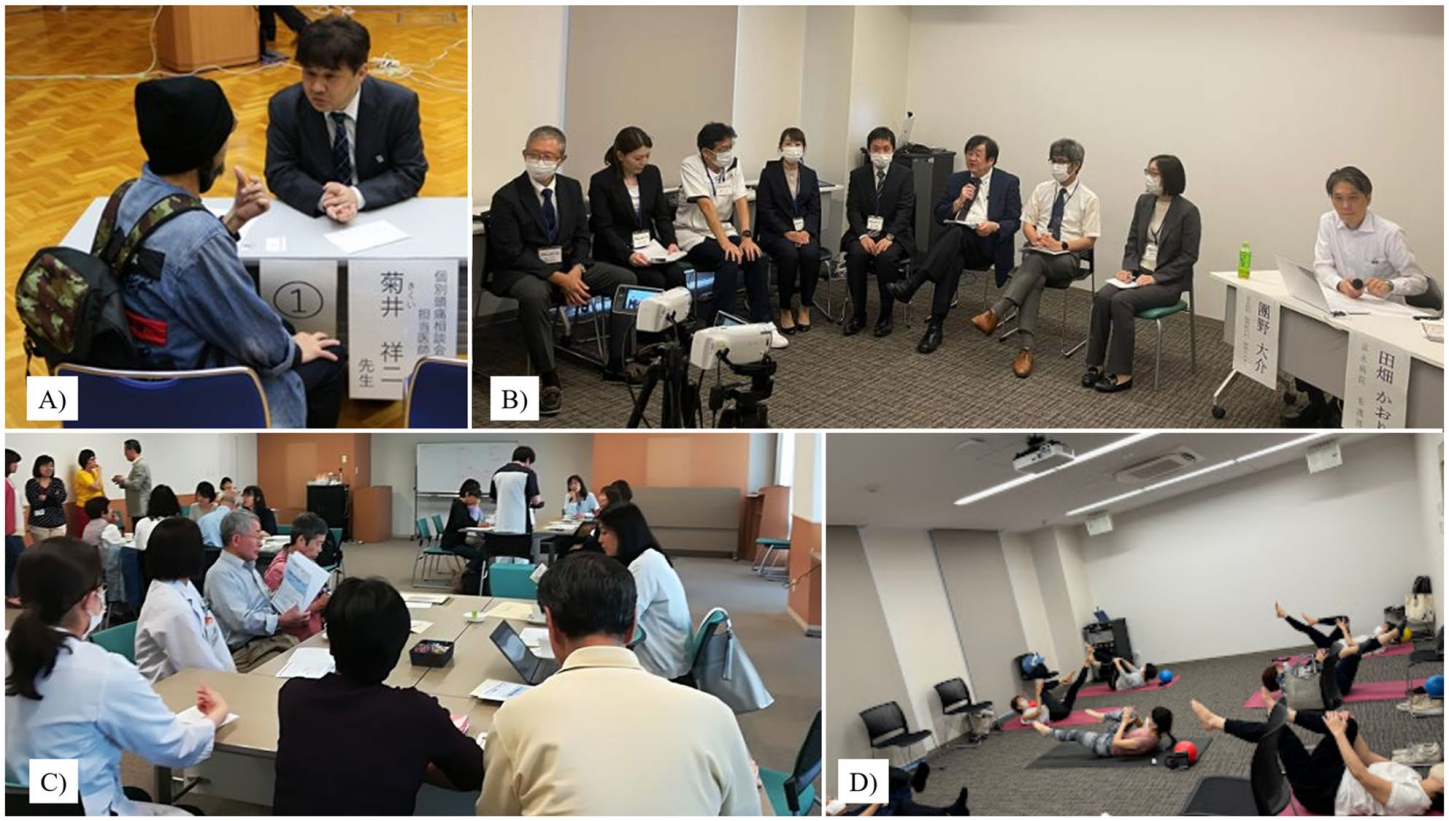
Promoting Patient Engagement through Diverse Educational Activities. Individual medical consultation sessions were conducted as part of the “Delivery Headache Class” held in Kanazawa City (**A**). A question-and-answer session following a headache education class at the Tominaga Hospital Headache Center fostering interactive dialogue among participants (**B**). A roundtable discussion session was held at Saitama International Headache Center (**C**). A complementary Pilates program was implemented to support headache management through physical activity (**D**)

Professor Takeshima, director of the Tominaga Hospital Tertiary Headache Center, proposed the “Delivery Headache Class” initiative. The sites for the “Delivery Headache Class” were selected in consultation with local headache specialists, taking into account not only the low density of headache specialists in the prefecture but also practical factors such as venue availability and the feasibility of securing support staff. The inaugural session was held in Oita City, Oita Prefecture, followed by a second session in Kanazawa City, Ishikawa Prefecture. Both regions are considered underserved in terms of headache care, with a limited number of headache specialists (10 in Oita Prefecture and 14 in Ishikawa Prefecture), highlighting the need for targeted educational and advocacy initiatives. In both locations, lectures were delivered jointly by local headache specialists and JPAC members. Attendees, including patients with headache and their advocates, reported high levels of satisfaction with the individual medical consultation sessions provided after the lectures (Fig. 5A).

JPAC also conducts a range of patient-centered initiatives throughout Japan with the aim of promoting education and advocating for individuals living with headache disorders. In addition to conventional academic-style lectures, JPAC organizes Q&A sessions and round table discussions at multiple institutions (Fig. 5B and C). These interactive formats provide opportunities for patients to share their experiences of living with headache and explore strategies for self-care [20]. Furthermore, various complementary programs are being offered, such as migraine prevention exercises, yoga, and Pilates, to holistically support the well-being of patients with headache disorders (Fig. 5D).

### Reducing the stigma associated with primary headache disorders

The stigma surrounding headache disorders increases suffering and confusion, ultimately delaying both research and effective treatment. Patients often face prejudice from HCPs, lack of support from family, dismissive attitudes in the workplace, and insufficient funding for research from policymakers [20]. Furthermore, stigmatizing attitudes toward individuals with migraine are more prevalent among those who have close relationships with individuals who have migraine and among individuals who have experienced migraine. Efforts to reduce such stigma may empower individuals with migraine to advocate for themselves, access appropriate care, and obtain increased support for necessary accommodations and treatment [21].

**Fig. 6:**
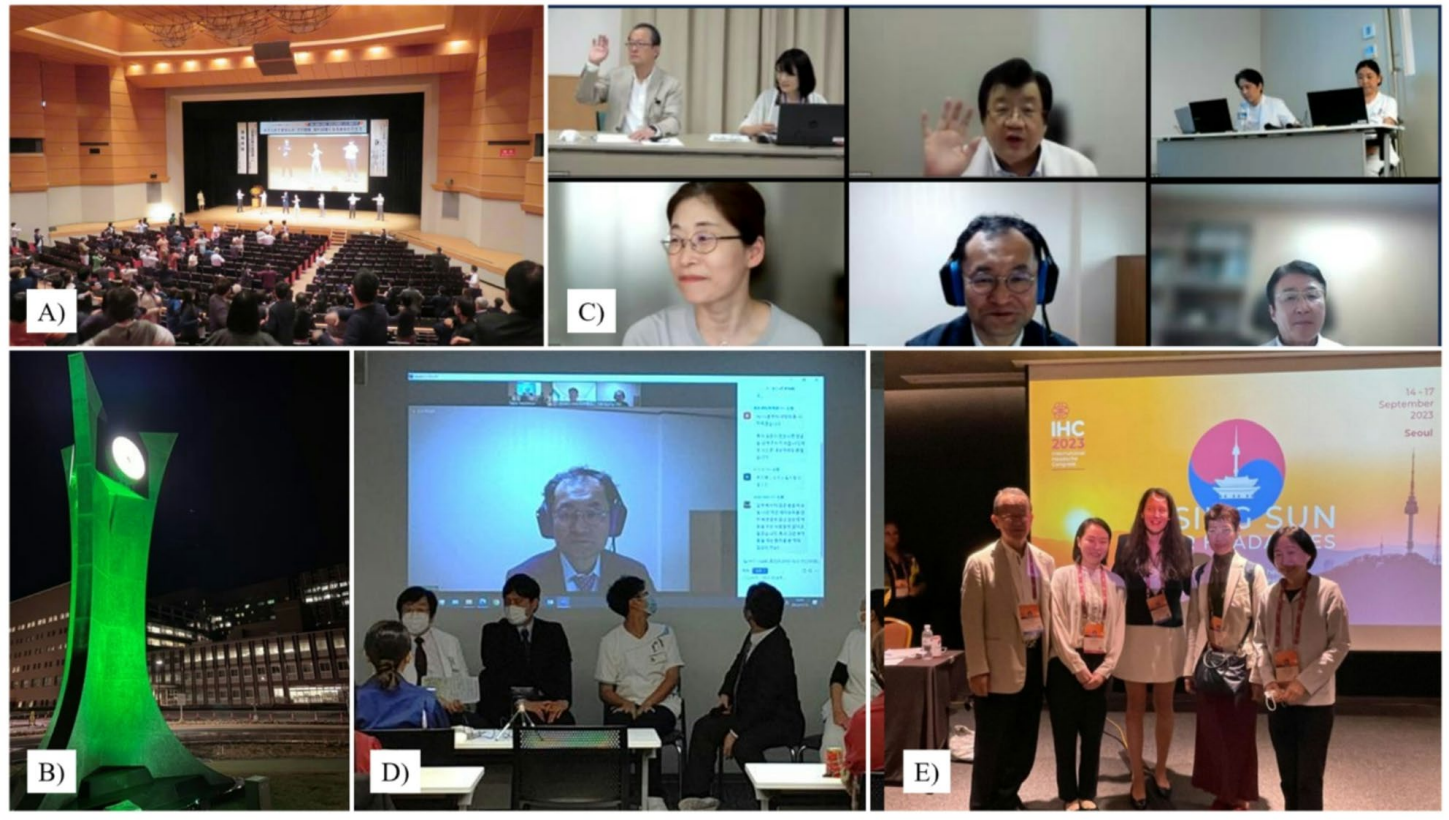
Reducing Stigma and Promoting Regional Collaboration in Asia. Following the commercial launch of CGRP-blocking medications, public interest in headache disorders has grown substantially, leading to increased attendance at public seminars (**A**). The “Green Light-up Campaign”: February 22 has been designated as “Headache Day,” during which a widespread call is made for public buildings and landmarks to be illuminated in green to raise awareness of headache disorders (**B**). The hybrid Japan–Korea Joint Headache Class, conducted both online and in person, was launched, enabling patients to share their experiences and perspectives with one another across language barriers (**C**, **D**). During the 2023 International Headache Congress in Seoul, a Japanese patient and a Korean patient each delivered presentations sharing their experiences at the GPAC Summit, marking a significant step forward in in-person regional collaboration in Asia (**E**)

JPAC has been actively involved in a range of public awareness seminars throughout Japan, by organizing, supporting, or delivering lectures, to enhance public understanding of headache disorders. Following the commercial launch of CGRP-blocking medications, public interest in headache disorders has grown substantially, resulting in increased attendance at these events (Fig. 6A). To promote awareness and reduce stigma, JPAC has been publishing online newsletters featuring photographs since May 2018. These newsletters highlight various JPAC activities and amplify the voices of individuals with headache disorders. As of May 2025, 65 issues have been published, significantly contributing to public awareness [22].

Another significant initiative undertaken by JPAC, the JHA, and JHS is the “Green Light-up Campaign.” A green light was selected because it is the least triggering wavelength of light in terms of migraine. February 22 has been designated as “Headache Day,” during which public buildings and landmarks are illuminated in green to raise awareness of headache disorders [23, 24]. Various facilities were illuminated in green, including university hospitals, local hospitals, clinics, TV and radio towers, clock towers, a prefectural government office, castles, a community welfare center, public halls, libraries, and convention centers (Fig. 6B). The JPAC team handed out green items, such as badges and ballpoint pens, and wore green items on the day, which has resulted in Headache Day recently gaining significant recognition [24].

One of the authors of this review, who is both a headache patient and a member of JPAC, reported that prior to her involvement in JPAC activities, she experienced substantial disruption in daily functioning and psychological distress due to headache. Through participation in JPAC activities, she gained awareness of efforts to raise public understanding of headache disorders, promote patient advocacy, and reduce stigma, which provided psychological support. She also became more engaged in learning about treatment and preventive strategies and actively participating in advocacy efforts. Through these activities, she shifted from a passive patient role to actively managing her condition and contributing to efforts that reduce stigma, which improved her functional capacity and overall well-being.

### Fostering asian regional collaboration and future perspectives

In response to the COVID-19 pandemic, an online headache education program was initiated, which subsequently evolved into a multicenter virtual headache class. Building on this foundation, the hybrid Japan–Korea Joint Headache Class Project was launched. The inaugural session was held in January 2023, followed by a second session in June of that same year.

The second session featured participation from healthcare professionals, including Professors Sakai, Takeshima, and Hirata, Nurse Tabata, and patients from the Tominaga Hospital Headache Center, Saitama International Headache Center, and Dokkyo Medical University Hospital in Japan. Professor Chu and a cohort of patients from Korea also participated in this event. In total, more than 10 HCPs and over 70 patients attended (Fig. 6C and D). These sessions were supported by volunteer interpreters, enabling patients to share their experiences and perspectives with one another across language barriers.

This initiative represents a significant step toward strengthening regional collaboration in Asia. The project also attracted attention from the Korean media, which covered the event with considerable interest and awe [25]. The Japan–Korea Joint Headache Class subsequently facilitated two patient presentations – one by a Japanese patient and another by a Korean patient – at the Global Patient Advocacy Summit (GPAS) held during the 2023 International Headache Congress in Seoul, which represented a significant step forward in promoting in-person regional collaboration in Asia (Fig. 6E).

In the future, JPAC aims to strengthen regional collaborations across Asia. By fostering a deeper understanding among patients and HCPs beyond national borders, JPAC and its collaborators can more effectively advance migraine awareness and patient advocacy.

### Quantitative and qualitative evaluation of JPAC activities

In this section, the outcomes of each JPAC program were examined using quantitative evaluation based on participant surveys and qualitative evaluation based on free-text comments, as well as coverage in media and social media. This approach enabled a comprehensive assessment of the multifaceted outcomes of JPAC activities from the perspectives of Reach and Engagement, Knowledge and Attitude Change, and System and Societal Impact. An overview of the quantitative and qualitative outcomes of the major JPAC programs is summarized in Table 2.

**Table 2:** Quantitative and qualitative outcomes of the Japanese patient advocacy coalition (JPAC) activities

Activity	Start Year	Participants and Quantitative Outcomes	Qualitative Insights and Observations
JPAC Annual Summit/Meeting	2017	12 participants (2017) → 37 (2025) 4 sponsoring companies (2025)	Patient, healthcare professional, and stakeholder engagement expanded. This shows growth in scale and diversity of participants.
Training Program for Headache Nurses	2019	50 participants (2019) → 200(2022) → 500(2023) → >500 (2024) 2022: Very useful 100%, Would participate again 89% 2023: Very useful 74%, Useful 24%, Would participate again 96%	Participants reported a deeper understanding of the role of nurses. Promotion of interdisciplinary collaboration and increased disease awareness were also observed.
Training Program for Hospital Staff	2024	39 participants (2024) → 42 (2025) Unaware of the importance of advocacy through multidisciplinary collaboration: 76% (2023) → 71% (2025) Importance of learning about headache: 76% (2023) → 80% (2025) Meaningful program: 86% (2023) → 86% (2025)	Participants reported increased awareness of headache education and program usefulness with emphasis on interdisciplinary collaboration.
Fujitsu Headache Project	2019	73,432 participants (90.5% of employees) Productivity increase: 14.7 days/year per employee with headache Annual cost saving: US$4,531 / Return on Investment (ROI) > 30 times	High participation; improved understanding of migraine; positive attitude change toward colleagues Reduced disability; increased productivity; workplace cost savings
Delivery Headache Class	2023	70 participants (2024)	Covered by community-based media; improved local awareness and understanding of headache and interdisciplinary collaboration.
Pilates Class	2022	274 participants (2022–2025, cumulative) 77% “Very satisfied”, 23% “Satisfied” Among participants who attended five or more sessions, Headache Impact Test-6 (HIT-6) scores decreased from 64.7 to 57.5, and Migraine Interictal Burden Scale-4 (MIBS-4) scores decreased from 8.7 to 4.3.	Participants reported positive behavioral expectations; increased activity, work, travel, hobbies, social interaction Suggested improvement in interictal daily functioning

### Reach and engagement

#### JPAC annual summit

The number of participants in the JPAC Annual Summit (formerly Meeting) increased from 12 in 2017 to 37 in 2025. In 2025, four sponsoring companies also participated, reflecting diversification of participants to include industry representatives. These results indicate that JPAC activities have expanded annually and evolved into a framework that engages not only patients and HCPs but also related stakeholders.

#### Training program for headache nurses

The Headache Nurse Training Program has been held four times, with participant numbers increasing from approximately 50 in 2019 to around 200 in 2022, 500 in 2023, and more than 500 in 2024. In 2024, participants came from at least 25 of the 47 prefectures, indicating expanded geographical reach. Survey responses indicated that in 2022, 100% of participants found the program very useful, and 89% would participate again; in 2023, 74% found it very useful, 24% found it useful, and 96% would participate again, demonstrating high engagement and educational value.

### Knowledge, attitude change, and behavioral change expectation

#### Pilates class

A total of 274 participants attended the Pilates Class over three years, with 77% reporting being “very satisfied” and 23% “satisfied.” Among participants who attended five or more sessions, scores on the Headache Impact Test-6 (HIT-6) decreased from 64.7 to 57.5, and scores on the Migraine Interictal Burden Scale-4 (MIBS-4) decreased from 8.7 to 4.3, suggesting improvement not only during migraine attacks but also in interictal daily functioning. Participants reported a variety of activities they wished to engage in upon improvement, including physical exercise, work, travel, hobbies, and social interactions, indicating positive changes in attitudes toward daily life and social activity, i.e., expectations for behavioral change.

#### Headache nurse training program

Free-text responses following the training indicated a deepened understanding of the role of nurses in headache care. Statements included, “As an outpatient nurse, I realized the importance of checking on patients’ daily life and feelings after consultations,” and “I understood that nurses have a role in listening to matters that patients find difficult to discuss with physicians.” These responses revealed shifts in recognition of the clinical process. Understanding of interdisciplinary collaboration also increased, with multiple responses such as, “I better understood the importance of team-based headache care” and “I recognized again the importance of interdisciplinary collaboration and want to share this on the ward.” Regarding disease understanding, reflective comments included, “I learned that the suffering caused by headaches is not fully understood” and “I realized that I had underestimated headaches without life-threatening risks,” suggesting early signs of behavior change prompted by learning.

#### Training program for hospital staff

Results from staff surveys indicated that implementation of this program reduced the proportion of staff reporting they were unaware of the importance of advocacy through multidisciplinary collaboration from 76% in 2023 to 71% in 2025. The proportion of staff recognizing the importance of learning about headache disorders increased from 76% in 2023 to 80% in 2025. Additionally, 86% of staff evaluated the program as meaningful in both 2023 and 2025.

### System and societal impact

#### Fujitsu Headache Project

In the Fujitsu Headache Project, participants were able to receive online consultations regarding their headache disorders. As a result, productivity increased by 14.7 days per year per employee with headaches, corresponding to an annual cost saving of USD 4,531 per employee with moderate to severe headaches. The positive return on investment (ROI) exceeded 30-fold, demonstrating substantial positive outcomes [17].

#### Delivery Headache Class

The Delivery Headache Class, implemented to promote patient education and advocacy in areas with few headache specialists, was featured in community-based online media with accompanying photographs. This coverage made the activities visible to local residents beyond the participants and serves as an indirect indicator of the recognition of these activities within the community. These events have the potential to build regional momentum for patient advocacy and are expected to support the sustained development of future advocacy initiatives.

#### “Headache Day” green light-up campaign

This initiative began in 2022 on Headache Day (February 22), following Prof. Hashimoto’s landmark illumination of the clock tower of Kumamoto University Hospital in green. In 2023, five facilities, including Kumamoto Castle, were illuminated [24]. This illumination of Kumamoto Castle served as a symbolic case to enhance social recognition of headache disorders and became an important turning point in the subsequent expansion of the campaign.

**Fig. 7:**
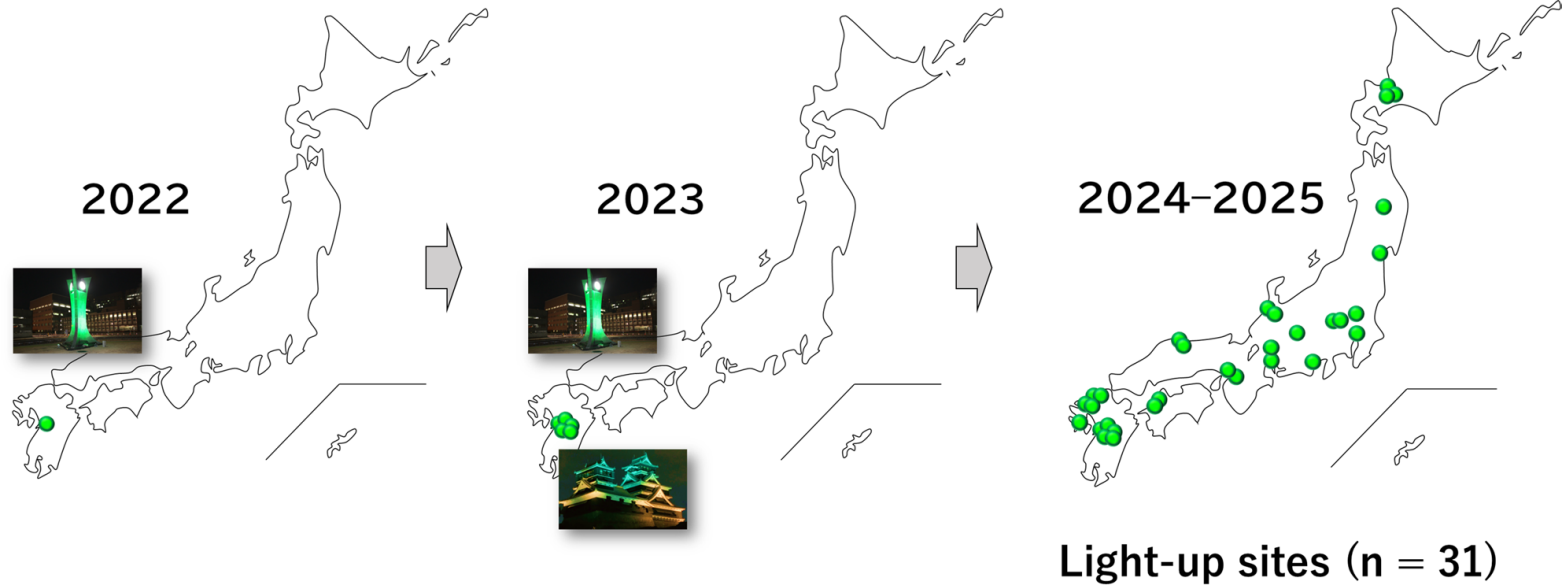
Geographical and Temporal Expansion of the “Headache Day” Green Light-Up Campaign (2022–2025). The campaign began in Kumamoto Prefecture in 2022, expanded to five sites including Kumamoto Castle in 2023, and reached nationwide implementation during 2024–2025, encompassing 31 sites across 19 prefectures. Expansion beyond medical institutions to symbolic landmarks such as castles, public facilities, and a railway station suggests successful penetration into local communities and broad societal impact. Green dots indicate individual light-up sites

From 2024 onwards, the initiative was implemented nationwide. Between 2024 and 2025, green light-up events were held in 31 sites across 19 prefectures on Headache Day. Cooperation from symbolic regional facilities, including castles, towers, a train station, and public institutions, facilitated the penetration of headache awareness into local communities. Moreover, coverage on social media further enhanced public awareness, suggesting broad societal impact (Fig. 7).

### Lessons learned

#### Successes

The success of JPAC is attributable to the implementation of patient-centered, multidisciplinary educational initiatives that enhance public awareness and promote advocacy for individuals with headache disorders. The integration of hybrid delivery formats facilitated broader accessibility, while structured training programs for HCPs and workplace-based interventions strengthened practical support. Furthermore, international collaboration has amplified these efforts, contributing to ongoing efforts to reduce headache-related stigma.

#### Challenges

The challenges faced by JPAC include the limited dissemination of workplace programs and insufficient educational opportunities for HCPs, including headache nurses. Securing stable funding and human resources is also essential. Additionally, although international collaboration, particularly within the Asian region, is expanding, overcoming language barriers and operational difficulties is imperative to establish a sustainable and effective cooperative framework. Furthermore, one of the most significant challenges remains the identification and implementation of appropriate outcome measures for advocacy and educational activities. Developing a comprehensive evaluation framework that effectively combines both quantitative methods (e.g., surveys and institutional data management) and qualitative approaches (e.g., interviews and focus groups with patients and stakeholders) is essential. Longitudinal follow-up over a period of years, assessing changes in healthcare utilization and employment status, can also help evaluate the sustained impact of these initiatives. Furthermore, publishing these findings in the academic literature will provide empirical evidence supporting the effectiveness of advocacy and contribute to the long-term sustainability of such efforts.

#### Areas for optimization and future directions

Expanding legislative advocacy to strengthen systemic support and promote changing workplace mindsets remains essential. Additionally, transitioning from a healthcare HCP–centered framework to one that enables patients to take on more active leadership roles is necessary. The utilization of advanced technologies, such as AI simultaneous interpretation, to overcome language barriers and enhance international collaboration is also important. Future directions should focus on further deepening patient-centered advocacy and fostering active patient participation across diverse sectors. By collaborating with workplaces, schools, media, and policymakers, efforts to improve public awareness and reduce stigma associated with headache disorders can be advanced. Moreover, strengthening international cooperation, particularly within the Asian region, will enable JPAC to continue serving as a global model for effective advocacy and educational initiatives.

### GPACH summit and headache master school Japan in Asia 2026

Against the backdrop of these challenges and strategic priorities, JPAC, in collaboration with GPACH and the JHS, plans to hold the “GPACH Summit and Headache Master School Japan (HMSJ) in Asia 2026,” a two-day meeting in Osaka, Japan, in November 2026, as a practical platform for patient-centered advocacy and education.

The meeting will bring together a wide range of stakeholders—including headache specialists, HCPs, social scientists, government officials, and industry representatives—to promote patient-centered advocacy and education and foster mutual understanding.

The expected outcomes of this meeting are as follows:

1. Reduction of regional disparities: By employing indicators such as the geographical distribution of participants and the regional implementation status of headache care support activities, the current status of interregional disparities can be assessed and measures to address them can be promoted.
2. Establishment of new funding frameworks: Collaboration with industry and government to secure sponsorships and grants will strengthen the sustainability of patient-centered activities.
3. Promotion of legislative and policy support: Developing concrete roadmaps for policy proposals and legislative improvements, engaging in discussions with stakeholders, and adopting declarations or official proposals at the GPACH Summit will contribute to the visibility and public dissemination of patient-centered activities.
4. In patient-centered support initiatives, linguistic differences among participants—including patients, HCPs, and other stakeholders—may impede effective communication. Accordingly, the pilot implementation of novel technologies, such as artificial intelligence (AI)–assisted simultaneous interpretation, is expected to facilitate seamless information exchange.

Building on efforts to address these structural challenges, these outcome domains will enable the quantitative and qualitative evaluation of JPAC’s impact from 2026 onward and will inform follow-up workshops and region-specific implementation plans aimed at the continuous improvement and expansion of activities. In line with the principle of “Think globally, act locally,” the meeting will promote practices rooted in local communities while maintaining a global perspective.

## Conclusions

Headache disorders, including migraine, remain underdiagnosed, undertreated, and highly stigmatized. Patient-centered advocacy with close collaboration between patient advocacy organizations such as JPAC and HCPs such as JHS and leading headache centers including the Tominaga Hospital Tertiary Headache Center, is key to moving the needle.

This review provides a comprehensive overview of JPAC’s history and collaboration with national and international initiatives of the JHS and JPAC, highlighting the growing momentum in raising awareness and promoting patient advocacy in Japan. With over 50 years of history and more than 3,400 members, the JHS is actively advancing diversity and internationalization. The JHS has also placed significant emphasis on promoting JPAC’s activities. The sustained efforts of JPAC are dynamic and have been well accepted by both patients with headache disorders and HCPs.

JPAC addresses the issues of migraine underdiagnosis, undertreatment, and stigma by raising awareness and providing education to everyone (public, workplace, HCPs, nurses, hospital staff, patients, caregivers, and occupational physicians) and by building capacity, including in underserved areas. Providing headache education to everyone at all levels is crucial to make a significant impact. In collaboration with the JHS and GPACH, JPAC is the leader in terms of headache advocacy in the workplace.

JPAC has also successfully implemented many of the actions recommended for headache advocacy in the Intersectoral Global Action Plan on Epilepsy and other Neurological Disorders (IGAP) toolkit. These include training the healthcare workforce, including HCPs, on headache disorders; providing headache education at the population level; and supporting patients by empowering them to take an active role in their headache care, including lifestyle changes [18]. Moving forward, it is essential to build on and extend the momentum generated by JPAC’s local, regional, and global initiatives, with special emphasis on workplaces, schools, the media, and policymakers to further enhance patient advocacy.

## Data Availability

No datasets were generated or analysed during the current study.

## Abbreviations

AI: Artificial intelligence
CGRP-mAbs: Calcitonin Gene-Related Peptide Monoclonal Antibodies
DALYs: Disability-Adjusted Life Years
GPAC: Global Patient Advocacy Coalition
GPACH: Global Patient Advocacy Coalition for Headache
GPAS: Global Patient Advocacy Summit
HCPs: Healthcare Professionals
HIT-6: Headache Impact Test-6
HMSJ: Headache Master School Japan
IGAP: Intersectoral Global Action Plan on Epilepsy and other Neurological Disorders
IHS: International Headache Society
IHS-GPAC: International Headache Society Global Patient Advocacy Coalition
JHA: Japan Headache Association
JHS: Japanese Headache Society
JPAC: Japanese Patient Advocacy Coalition
MIBS-4: Migraine Interictal Burden Scale-4
MIDAS: Migraine Disability Assessment Total Score
MiRS: Migraine-Related Stigma
QOL: Quality of Life
ROI: Return on Investment
WHO: World Health Organization
